# Validation of the breathlessness, cough and sputum scale to predict COPD exacerbation

**DOI:** 10.1038/npjpcrm.2016.83

**Published:** 2016-12-01

**Authors:** Rebecca DeVries, David Kriebel, Susan Sama

**Affiliations:** 1 Department of Work Environment, University of Massachusetts Lowell, Lowell, MA, USA

## Abstract

The breathlessness, cough and sputum scale (BCSS) is a three-item questionnaire rating breathlessness, cough and sputum on a 5-point Likert scale from 0 (no symptoms) to 4 (severe symptoms). Researchers have explored the utility of this tool to quantify efficacy of treatment following a chronic obstructive pulmonary disease (COPD) exacerbation; however, little work has been done to investigate the ability of the BCSS to predict COPD exacerbation. As part of a prospective case-crossover study among a cohort of 168 COPD patients residing in central Massachusetts, patients were asked standard BCSS questions during exacerbation and randomly identified non-exacerbation (or healthy) weeks. We found that the BCSS was strongly associated with COPD exacerbation (OR=2.80, 95% CI=2.27–3.45) and that a BCSS sum score of 5.0 identified COPD exacerbation with 83% sensitivity and 68% specificity. These results may be useful in the clinical setting to expedite interventions of exacerbation.

## Introduction

Research on improving the life course and treatment of COPD requires reliable methods for defining an exacerbation. Researchers have used a wide variety of definitions based on discrete events (such as emergency department visits or increased medication usage) and self-reported respiratory symptoms, but few studies have compared the utility of different approaches.^1^ Symptom-based exacerbation definitions have several potential benefits, but there is little consensus on which algorithm is the most useful.^1,2^ As part of a prospective case-crossover study evaluating potential risk factors for COPD exacerbation, we gathered self-reports of respiratory symptom severity among a cohort of COPD patients using the breathlessness, cough and sputum scale (BCSS).^3^ The BCSS is a sum of responses to three questions rating breathlessness, cough and sputum on a 5-point Likert scale from 0 (no symptoms) to 4 (severe symptoms). Although the BCSS score has been used to assess efficacy of treatment following a COPD exacerbation in clinical trials,^4–6^ little work has been done to evaluate its ability to predict an imminent exacerbation, which could have important implications for future observational studies as well as disease management. In fact, Pauwels *et al.*^1^ specifically called for a comparison of exacerbation-related symptom changes to random variations outside clinically defined exacerbation periods. This brief communication provides a comparison of BCSS reports for 168 COPD patients at the start of an exacerbation and during non-exacerbation periods. With these data, we were able to calculate the sensitivity and specificity of specific values of the BCSS as a prognostic tool for COPD exacerbation.

## Methods

We conducted a prospective case-crossover study among a cohort of COPD patients drawn from a disease management group managed by a large group medical practice in central Massachusetts. During a 15-month study period (January 2011–March 2012), COPD patients enrolled in the disease management group were instructed to call clinic nurses when, following their prescribed disease management group treatment plan, they were developing worsening symptoms and thought it was time to begin using pre-filled medications. The disease management group nurse confirmed or disagreed with their assessment based on telephone interview and her clinical judgement. If the exacerbation was confirmed and the nurse agreed that medications were needed, she asked a series of questions concerning the patient’s respiratory symptoms, daily living behaviours and activities in the previous week. The standard BCSS questions were a part of this questionnaire.^3^ The same questionnaire was also administered via telephone at up to three randomly identified times when the patient was not experiencing exacerbation symptoms to evaluate symptom scores and exacerbation risk factors during normal healthy weeks. All study materials and protocols were approved by Reliant Medical Group Institutional Review Board and informed consent was obtained from all patients.

The association between BCSS and exacerbation risk was estimated using conditional logistic regression, which maintains the match between each individual participant’s exacerbation and non-exacerbation risk factor data. ROC curves were developed to identify an optimal cut-point to discriminate between exacerbation and non-exacerbation weeks.^6^ ROC curves cannot be estimated conditional on participant and so the match was necessarily dropped. The results of investigations of environmental risk factors for COPD exacerbation using these data are described in two previous papers—a case-crossover study of air pollution^7^ and a cross-sectional study of worsening symptoms from indoor chemical exposures.^8^

## Results

The sample population included 168 COPD patients, contributing information to 231 exacerbation and 389 non-exacerbation periods. The participants were predominantly white (97%), over the age of 65 (75%), and with severe to very severe COPD (based on GOLD classification^9^), 50% and 18%, respectively. Nearly half (43%) had received a doctor diagnosis of asthma in addition to their COPD diagnosis. Sixty-five percent of participants experienced one exacerbation over the study period, whereas 30% experienced two exacerbations and 5% experienced three exacerbations. Each participant also provided data on an average of 2.3 (s.d.=0.59) ‘control’ or healthy periods with which the exacerbation onset data were compared.

The BCSS was strongly associated with risk of exacerbation. The odds ratio (OR) was 2.80 (95% CI: 2.27–3.45), suggesting that each one point increase in the score increased the risk of exacerbation by 180%. Although the BCSS as published consists of a sum of the three different symptoms scores (breathlessness, cough and sputum), we disaggregated the score and evaluated the risk of exacerbation associated with increases in each of the three components separately. The objective was to see whether one symptom was more strongly associated with exacerbation risk than the others. The results showed similar risks estimates for each of the three different symptoms; the ORs were 3.14 (95% CI: 2.21–4.48) for breathlessness, 2.62 (95% CI: 1.80–3.83) for cough and 3.20 (95% CI: 2.08–4.92) for sputum. This suggests that it is not necessary to weight any one symptom more heavily than another when computing a BCSS summary measure and that the simple sum of the three symptoms' self-reports is appropriate.

**Figure 1:**
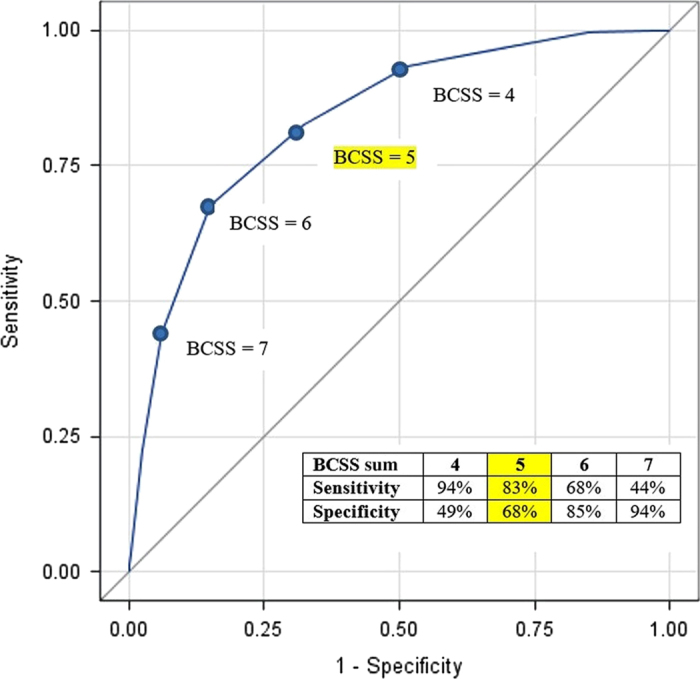
ROC curve for BCSS sum score and COPD exacerbation.

We developed a receiver operating characteristic curve (ROC curve) comparing true-positive rates to false-positive rates to quantify how well the BCSS predicted COPD exacerbation.^10^ The area under the curve for this ROC curve was estimated at 0.84, indicating good predictive ability (Figure 1). As each point on this ROC curve represents a sensitivity/specificity pair corresponding to a particular decision cut-point,^10^ we identified a BCSS of 5.0 as the best threshold to identify signs of COPD exacerbation (Figure 1). Using a score of 5.0 as a cutoff, the BCSS will identify an exacerbation with 83% sensitivity and 68% specificity. If one wanted to be more conservative when detecting COPD exacerbation, they could select a lower BCSS cutoff of 4.0. This would increase sensitivity to 94% but cause specificity to drop considerably (49%), resulting in more false positives. On the other hand, the BCSS could be optimised to improve ability to identify non-exacerbation periods by selecting a value that increases specificity. A BCSS threshold of 6.0, for example, would identify an exacerbation with 68% sensitivity and 83% specificity.

The mean increase in BCSS between exacerbation and non-exacerbation weeks was +2.57 (s.d.=1.95). This increase was slightly larger among those with mild to moderate COPD as compared with those with severe to very severe COPD (mean difference of +3.02 (s.d.=2.09) versus mean difference of +2.41 (s.d.=1.84), *P*=0.06).

## Discussion and Conclusion

Although individual COPD patients varied widely in their symptoms of breathlessness, cough and sputum, we found that a BCSS of 5.0 was a good predictor of risk of a clinically confirmed exacerbation. Leidy *et al.*^3^ reported a very similar BCSS score (5.29) for COPD patients in the 7 days before an exacerbation (the same time window that we looked at). Freeman *et al.*^11^ reported fairly similar changes in BCSS in their study of COPD patients between ‘stable’ clinic visits when the median BCSS was 3.5, increasing to 8.0 during acute exacerbations. The sample size in Freeman’s study was small, however, and there was no intention to try to validate the BCSS.

The 2.57-point mean increase in BCSS when going from normal periods to exacerbations in our data agrees well with the decreases in BCSS reported in three clinical trials of COPD treatments. Improvements of BCSS of −1.3 (3), −1.1 (5) and −1.9 (4) were associated with effective treatments.

These findings on the accuracy of BCSS to predict clinically confirmed exacerbations are especially important as research continues to document that objective measures (such as changes in pulmonary function) do not correlate well with subjective measures (such as changes in COPD symptoms).^1,2^ We conclude that the use of a simple three-item questionnaire may provide an easy and accurate way to identify patients who are at risk of exacerbation to expedite interventions of COPD exacerbation.

## Funding

U.S. National Institute for Environmental Health Sciences R21-ES017849 and U.S. National Institute for Occupational Safety and Health T01-OH008424.
